# Protease Inhibitors Protect Bovine Colostrum or Chicken Egg Growth Factors from Pancreatic Enzyme Digestion in AGS Cells or Colitic Rats

**DOI:** 10.1093/jn/nxab197

**Published:** 2021-06-16

**Authors:** Tania Marchbank, Sandra J M ten Bruggencate, Raymond J Playford

**Affiliations:** Centre of Immunobiology, Blizard Institute, Barts and The London School of Medicine, Queen Mary, University of London, London, United Kingdom; Research and Development, Pantheryx, Inc., Boulder, CO, USA; Centre of Immunobiology, Blizard Institute, Barts and The London School of Medicine, Queen Mary, University of London, London, United Kingdom; Research and Development, Pantheryx, Inc., Boulder, CO, USA

**Keywords:** nutraceuticals, gut repair, growth factors, injury, epidermal growth factor, soya bean trypsin inhibitor, ovomucoid, ulcerative colitis, inflammatory bowel disease

## Abstract

**Background:**

Bovine colostrum (BC) and chicken egg contain proteins possessing growth factor activity. Epidermal growth factor (EGF) provides much of the pro-reparative activity within BC. Clinical use of orally administered peptide growth factors is hampered by digestion from pancreatic proteases.

**Objectives:**

We examined whether adding a protease inhibitor [soybean trypsin inhibitor (SBTI) or ovomucoid] protected bioactivity of BC ± egg or EGF alone against pancreatic digestion using in vitro and in vivo models.

**Methods:**

BC, egg, or EGF alone or in combination with trypsin inhibitors were tested for proliferative (Alamar blue) activity using human gastric adenocarcinoma (AGS) cells, prior to and after incubation with HCl/pepsin and trypsin/chymotrypsin. Data were analyzed using 2-factor ANOVA. Eight groups (*n* = 10) of adult female Sprague-Dawley rats (mean: 188.3 ± 0.8 g) received 20 mg/kg/d of BC + egg, 100 μg/d of EGF, 5 mg/d ovomucoid, or 10.8 mg/d SBTI, alone or in combination (in 1 mL 3% NaHCO_3_) by gavage for 9 d and dextran sodium sulfate (DSS; 5% in drinking water) for the final 7 d. Histology, microscopic damage score, and myeloperoxidase (MPO) were assessed and analyzed using 1-factor ANOVA.

**Results:**

Proliferative activities of BC, egg, or EGF were reduced 40–57% by HCl/pepsin exposure and further reduced 14–24% by chymotrypsin/trypsin. Co-addition of SBTI or ovomucoid truncated the decrease in proliferative bioactivity caused by chymotrypsin/trypsin by 54–100% (*P* < 0.01). In vivo study showed oral EGF alone or protease inhibitors given alone were ineffective in reducing DSS damage, whereas SBTI with EGF or ovomucoid with BC + egg improved protective effects on weight gain, disease activity score, colonic MPO, and histology damage by 3–4-fold (*P* < 0.01).

**Conclusions:**

Studies using AGS, cells, and Sprague-Dawley rats showed the protease inhibitors ovomucoid and SBTI protected BC, egg, and EGF against loss of bioactivity due to pancreatic enzymes and, when given with NaHCO_3_, enhanced colonic protection against DSS damage.

## Introduction

Inflammatory bowel disease (IBD) is a severe, relapsing condition predominantly affecting the gastrointestinal tract and is subdivided into ulcerative colitis (UC) affecting the large intestine and Crohn's disease, which may affect the entire gastrointestinal tract, with a predilection for the terminal ileum. In both conditions, an excess inflammatory response leads to disruption of mucosal integrity, ingress of luminal contents into the mucosa and bloodstream, and further exacerbation of the inflammatory process. Current therapies involve powerful immune modulators with serious side effects. Therapies include prednisolone, azathioprine, or monoclonal antibodies directed against the inflammatory cascade (e.g., TNF-α) with associated risks of serious infections and/or bone marrow suppression (1). Although primary nutritional therapy to treat UC is not clinically effective, there is stronger evidence for the value of elemental or polymeric diets for treating active Crohn's disease, especially in children (2). However, restriction of normal food intake and replacement with relatively unpalatable diets is far from optimal and addition of an effective nutritional supplement that can be used alongside a normal diet has obvious advantages.

Partly because of concerns over side effects of pharmaceutical agents, there is a demand from the public for more “natural” types of products, many of which fall under the category of nutraceuticals. Nutraceuticals, also known as functional foods, are products derived from food sources that provide extra health benefits, in addition to their basic nutritional value. Bovine colostrum (BC) provides a strong example of an evidence-based nutraceutical, with >6000 preclinical and clinical studies having been published. Colostrum is the first milk produced during the initial few days after birth. Compared with the milk subsequently produced, it is rich in immunoglobulins, antimicrobial peptides, and growth factors (3). BC is a side product of the dairy industry and has been shown to have potential value for the prevention and treatment of a variety of gastrointestinal conditions such as nonsteroidal anti-inflammatory drug (NSAID)–induced gut injury (4, 5).

Evidence in support of the use of BC for IBD includes findings that oral BC is beneficial in reducing trinitrobenzenesulfonic acid (TNBS) or dextran sodium sulfate (DSS)–induced colitis in mice (6,7) and additional synergistic benefits were seen using the DSS model if BC was co-administered with uncooked egg powder, an additional source of antibodies and growth factors (7). EGF and the EGF receptor (EGFR) play important roles in mediating the effects of BC and egg, with high levels of EGF and transforming growth factor (TGF)-α being present in BC (8), and the addition of an EGFR blocker removing the majority of pro-proliferative and reparative activity of BC and egg (7, 8).

We previously reported an initial clinical trial using BC enemas for distal colitis that showed positive results with reduced symptoms and inflammation (9). However, if BC is to be beneficial in patients with UC that extends more proximally than the left side of the colon (i.e., beyond the reach of the enema therapy) or for patients with Crohn's disease (where small intestinal damage predominates), oral administration is required. However, BC (and egg proteins if co-administered) would be susceptible to at least partial digestion by gastric and pancreatic enzymes, reducing the effective dose delivered to the distal bowel. Peptic digestion in the stomach is easily overcome by increasing the pH >4 (10), whereas protection against pancreatic enzyme digestion is more problematic.

We hypothesized that co-administration of protease inhibitors may be beneficial to preserve the bioactivity of ingested growth factors of BC and/or egg within the small intestine and selected ovomucoid and soybean trypsin inhibitor (SBTI), as they are both serine protease inhibitors naturally found in food products (11, 12). We initially examined the ability of ovomucoid and SBTI to preserve proliferative bioactivity of BC and egg alone and in combination and also examined the stability of EGF tested in isolation as it is one of BC's most important active constituents for reparative function (3). We then progressed to an in vivo rat DSS-induced colitis model to examine whether ovomucoid or SBTI given alone could truncate DSS-induced injury (through preserving endogenous luminal growth factors) and whether they could enhance the protective effect of orally administered BC + egg or EGF.

## Methods

### BC, egg, EGF, and protease inhibitor samples

Pasteurized BC powder (ColostrumOne^TM^) and a commercial chicken whole egg powder were provided by Pantheryx, Inc. (Boulder, CO, USA). The BC was collected during the first 24 h post-calving as there is a rapid decline in IgG and other bioactive constituents after this time (8). The BC powder comprised 48.32 g protein, 16.25 g fat, 24.53 g carbohydrate, 6.4 g ash and 4.5 g moisture, and 15g IgG per 100 g of powder. Egg powder comprised 50.45 g protein, 42.71 g fat, 1.25 g carbohydrate, 1.72 g moisture, and 3.87 g ash per 100 g. Sources of other peptides were as follows: human EGF (Peprotech), SBTI (Roche), ovomucoid (Sigma), and BSA (Sigma). The BC and egg combination were tested in the in vitro and in vivo studies in a ratio of 60:40, based on the beneficial synergistic effects against DSS injury demonstrated by us previously (7).

Trypsin inhibitor activity of test compounds was assessed in triplicate using a standard Na-benzoyl-DL-arginine-*p*-nitroanilide (BAPNA) trypsin inhibition assay based on the method of Erlander et al. (13) and expressed as mean ± SEM. Amounts of trypsin inhibitor activity of test product were as follows: BC, 7.41 ± 0.44 μg trypsin inhibited/mg product; egg, 42.93 ± 4.41 μg trypsin inhibited/mg product; EGF, 1.43 ± 0.12 μg trypsin inhibited/mg product; ovomucoid, 703 ± 7.53 μg trypsin inhibited/mg product; and SBTI, 838 ± 8.94 μg trypsin inhibited/mg product.

### Cell line

Human gastric adenocarcinoma (AGS) cells are derived from gastric adenocarcinoma of a 54-year-old female (American Type Culture Collection, LGC Standards, UK) (14).

### Study 1. Effect of protease inhibitors on growth factor preservation in vitro

#### Digestion of samples

**FIGURE 1:**
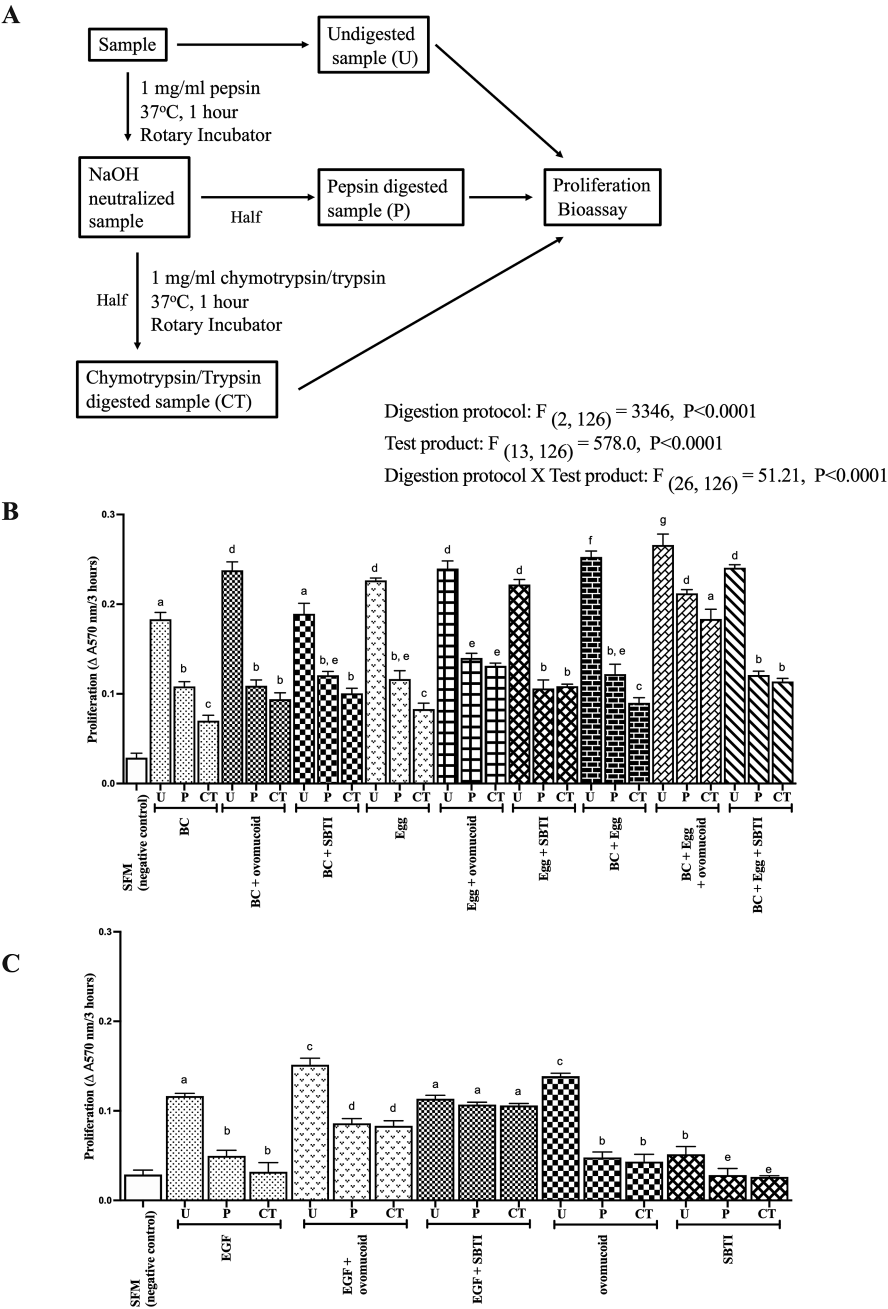
Effect of the protease inhibitors SBTI and ovomucoid on growth factor preservation in vitro. (A) BC, egg, EGF, ovomucoid, and SBTI were exposed to the digestion protocol shown. Pro-proliferative activity (change in absorbance at 570 nm assessed 3 h after adding Alamar blue) of aliquots of undigested samples (U) were compared against results following HCl/pepsin (P) and chymotrypsin + trypsin (CT) digestion. (B, C) Results of samples tested alone and in combination. Values are means ± SEM of triplicate independent well measures. Data analyses by 2-factor ANOVA used test product and digestion exposure as factors, with post hoc Tukey's multiple-comparison test used to compare means between the groups. Means that do not have a common letter (a–g) are significantly different from each other, *P* < 0.01. Comparisons discussed in the main text relate to differences between “U”, “P”, and “CT” samples within each test product and comparing differences between the test products treated in an identical fashion. BC, bovine colostrum; EGF, epidermal growth factor; SBTI, soybean trypsin inhibitor; SFM, serum free medium.

To reproduce intraluminal exposure of peptides within the stomach and small intestine, samples were tested undigested ("U" samples), following exposure to HCl/pepsin alone (1 h, "P" samples) or HCl/pepsin (1 h) followed by chymotrypsin and trypsin exposure (1 h, "CT" samples), using the protocol shown in **Figure 1**A. Briefly, aliquots of BC alone (400 mg),  egg alone (400 mg),  BC + egg (400 mg total weight in 60:40 ratio), and EGF (100 μg) were made up in 10 mL PBS in the presence or absence of ovomucoid (500 mg) or SBTI (1 g). Samples were then incubated at 37°C without the addition of pepsin, chymotrypsin, or trypsin (undigested controls "U") or incubated in pepsin (1 mg/mL) pH 2 in a rotary incubator at 37°C for 1 h followed by neutralization to pH 7 using NaHCO_3_. Half of the aliquot was then removed (HCl/pepsin-digested sample, "P") and the remainder incubated in chymotrypsin and trypsin (1 mg/mL) for 1 h (chymotrypsin/trypsin-digested sample, "CT"). Additional samples examined the susceptibility of ovomucoid or SBTI alone to digestion. All samples were subsequently analyzed for bioactivity in the same cell proliferation assay.

#### Proliferation assays

Cell proliferation assays were performed as previously described (15) using Alamar blue (ThermoFisher Scientific), as per the manufacturer's instructions. BC + egg constituents were tested at 1 mg powder/mL, ovomucoid at 1.25 mg/mL, EGF at 0.25 μg/mL, and SBTI at 2.5 mg/mL. These concentrations were based on preliminary dose–response comparisons.

### Study 2. Rat DSS colitis model

#### Ethics

Animal studies were performed within BolderBIOPATH Laboratories, Boulder, Colorado, in accordance with the commercial test facility standard operating procedures and license, the WHO Quality Practices in Basic Biomedical Research guidelines, and in compliance with all state and federal regulations, including USDA Animal Welfare Act 9 CFR parts 1–3. Federal Register 39,129, July 22, 1993.

#### Protocol

Female Sprague-Dawley rats (174–204 g) were obtained from Envigo RMS, Inc. (Indianapolis, IN), housed in standard cages (5 per cage), and fed Envigo Teklad 8640 diet (Envigo Ltd.). This comprised 22% protein, 40.6% carbohydrate, 5.5% fat, 3.9% fiber, energy density of 3 kcal/g [8640 datasheet 0915.xls (envigo.com)], and tap water ad libitum. Rats were acclimated for 7 d prior to being placed in the study. Methods used were as described by us previously (7). Eight groups were tested (*n* = 10 per group). All rats received a single 1-mL oral gavage made up in 3% sodium bicarbonate to neutralize gastric acidity (16, 17) daily for 9 d. The negative control group received no DSS but received daily gavage with BSA (20 mg/kg, BSA). The positive control group received DSS and gavage with 20 mg/kg BSA. The remaining 6 groups received DSS plus 1 of the following treatments: BC + egg (60:40 ratio, 20 mg/kg), EGF (100 μg/dose), ovomucoid (5 mg/dose), or SBTI (10.8 mg/dose) alone. The final 2 groups comprised the combination of EGF + SBTI or BC + egg + ovomucoid. These groups were chosen based on the results of study 1, as optimal protective effects were seen with ovomucoid for BC + egg and SBTI for EGF.

Colitis was induced by adding 5% (wt:vol) DSS (molecular mass, 40–50 kDa; USA catalog no. DE136, lot no. 1IG1103; Spectrum Chemicals) to the drinking water for 7 d, starting from day 3 of the test product gavage period. Mean DSS and food consumption were noted per cage each day. No significant differences were seen in water intake (and therefore DSS) between the groups. Rats were weighed daily and visually inspected for signs of distress, diarrhea, and rectal bleeding. The disease activity index [DAI; based on Cooper et al. (18)] was assessed daily following induction of colitis. The DAI combines the scores of weight loss, stool consistency, and bleeding divided by 3. A cumulative score was then determined over the 7-d DSS treatment period.

At the end of the study, rats were anesthetized by CO_2_ inhalation and killed by bilateral thoracotomy, and colonic tissue was collected for biochemical and histopathological assessment. Microscopic damage was assessed using scoring system described by Williams et al. (19). Colonic tissue was also analyzed for myeloperoxidase (MPO) activity (used as a marker of neutrophilic infiltration) as described previously (20).

### Statistical analysis

All results are expressed as means ± SEMs. Statistical analyses were performed using GraphPad Prism 9 version 9.1.10. A Shapiro-Wilk test for normality of data showed equal variances between groups. Digestion study data were analyzed with a 2-factor ANOVA, using digestion protocol and test product as factors, with the program using Tukey's multiple-comparison test to compare means between groups. Significance of differences between groups was generated by the statistical program as part of the overall ANOVA analysis. In vivo studies were analyzed using the repeated-measures 1-factor ANOVA with treatment as a factor with Geisser-Greenhouse correction for sphericity option. With regard to the 2-factor ANOVA, the statistical program generates the statistical differences between groups as part of the overall ANOVA using Tukey's multiple-comparison test. Power calculation for the DSS rat model for *n* = 10, 10% change, with *P* < 0.05 showed a power of 0.9.

## Results

### Study 1. Effect of protease inhibitors on growth factor preservation in vitro

Similar results were seen using BC or egg alone; exposure to HCl/pepsin reduced proliferative bioactivity by ∼50%, with a further 18% reduction following CT exposure. Co-presence of ovomocoid or SBTI did not influence HCl/pepsin susceptibility but reduced CT digestion by ∼80% (Figure 1B; all *P* < 0.01).

BC + egg showed a similar susceptibility to HCl/pepsin and CT digestion as incubating the individual factors alone. Co-presence of SBTI did not affect peptic HCl/pepsin susceptibility but reduced CT sensitivity. Co-presence of ovomucoid reduced both HCl/pepsin and CT-induced loss of biological activity (Figure 1B). EGF incubated alone with HCl/pepsin caused a 76% reduction in proliferative bioactivity, with a further reduction of 20% following incubation with CT (Figure 1C). Co-presence of ovomucoid did not affect HCl/pepsin susceptibility but significantly reduced loss of bioactivity caused by CT. Co-presence of SBTI reduced both HCl/pepsin and CT loss of biological activity.

SBTI alone did not stimulate proliferation when given alone (Figure 1C). Ovomucoid alone stimulated proliferation, but this was completely lost following HCl/pepsin digestion (Figure 1C).

### Study 2. Rat DSS colitis model

#### Body-weight changes

**FIGURE 2:**
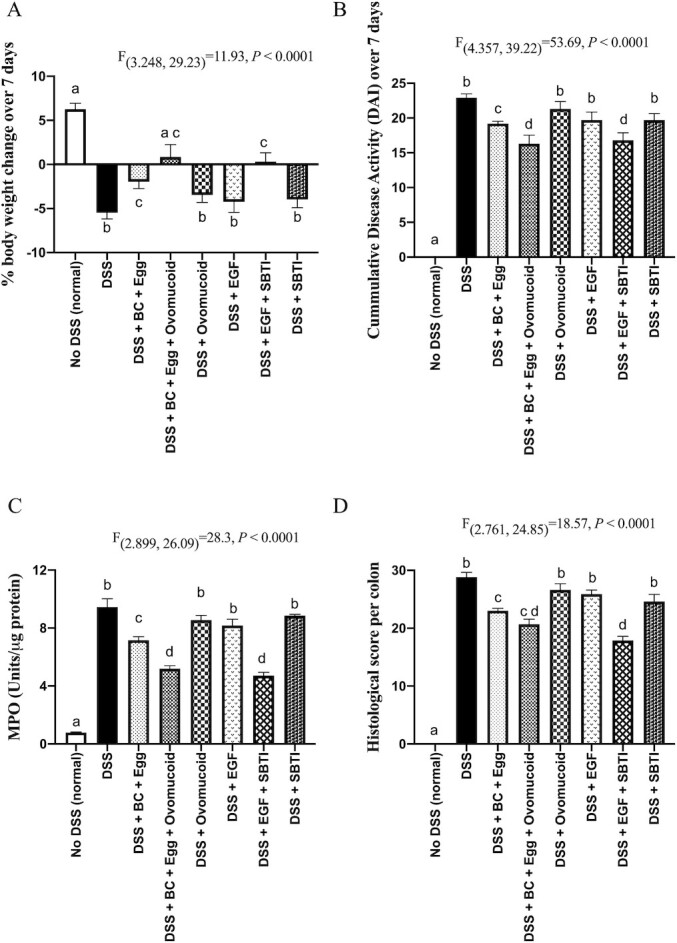
Influence of the protease inhibitors SBTI and ovomucoid on protective activity of BC + egg or EGF in DSS-induced colitis in rats. Rats (*n =* 10/group) received no DSS (normal) or DSS in drinking water for 7 d. Active treatment groups also received BC + egg ± ovomucoid or EGF ± SBTI for 9 d, starting 2 d prior to DSS. Control rats received BSA. The 1-mL gavage also contained 3% sodium bicarbonate to neutralize gastric acidity. (A) Cumulative total body-weight change over 9 d. (B) Cumulative DAI score (18). (C) Colonic tissue MPO concentrations. (D) Total histological colitis score (19). Values are means ± SEMs. Data analyses by 1-factor ANOVA with post hoc Tukey's multiple comparison test were used to compare means between the groups. Labeled means without a common letter differ, *P* < 0.01. Comparisons discussed with the main text relate to the efficacy of the various test products in reducing injury caused by DSS and in enhancing the efficacy of BC + egg or EGF alone. BC, bovine colostrum; DAI, disease activity index; DSS, dextran sodium sulfate; EGF, epidermal growth factor; MPO, myeloperoxidase; SBTI, soybean trypsin inhibitor.

Rats that did not receive DSS showed an increase of 11.8 ± 1.9 g over the 7 days (**Figure 2**A). In contrast, rats that received DSS alone showed marked weight loss (−10.2 ± 1.5 g over the 7 d, *P* < 0.01, compared with no DSS) over the same period. Administration of ovomucoid alone to DSS-treated rats did not significantly affect weight loss, whereas BC + egg significantly truncated weight loss induced by DSS (*P* < 0.01 vs. DSS alone). The most improvement in weight was seen in rats given BC + egg + ovomucoid together (*P* < 0.01).

EGF or SBTI given alone did not affect the weight loss induced by DSS. In contrast, the combination of EGF + SBTI showed marked improvement in weight compared with DSS alone (*P* < 0.01).

#### DAI score

Analyses of DAI scores gave similar results to those seen following weight changes of rats. Ovomucoid given alone did not affect DAI scores (Figure 2B). Beneficial effects were seen using BC + egg, and the best result was seen using the combination of BC + egg + ovomucoid (*P* < 0.01 vs. DSS alone or BC + egg in DSS-treated rats).

EGF or SBTI given alone did not affect DSS-induced changes in DAI, whereas the combination significantly reduced DAI scores (*P* < 0.01).

#### Colonic MPO concentrations

Results from MPO analyses were in keeping with body-weight changes and DAI scores (Figure 2C). Administration of DSS alone caused a 13-fold increase in colonic MPO. Administration of ovomucoid to DSS-treated rats alone did not improve MPO concentrations, whereas BC + egg truncated the increase in MPO concentrations caused by DSS (*P* < 0.01). Additional benefit was seen if the BC + egg was co-administered with ovomucoid (*P* < 0.01 vs. DSS alone, DSS + ovomucoid, or DSS + BC + egg).

EGF or SBTI given alone did not affect DSS-induced changes in MPO, whereas the combination significantly reduced MPO concentrations (*P* < 0.01).

#### Histological assessment

**FIGURE 3:**
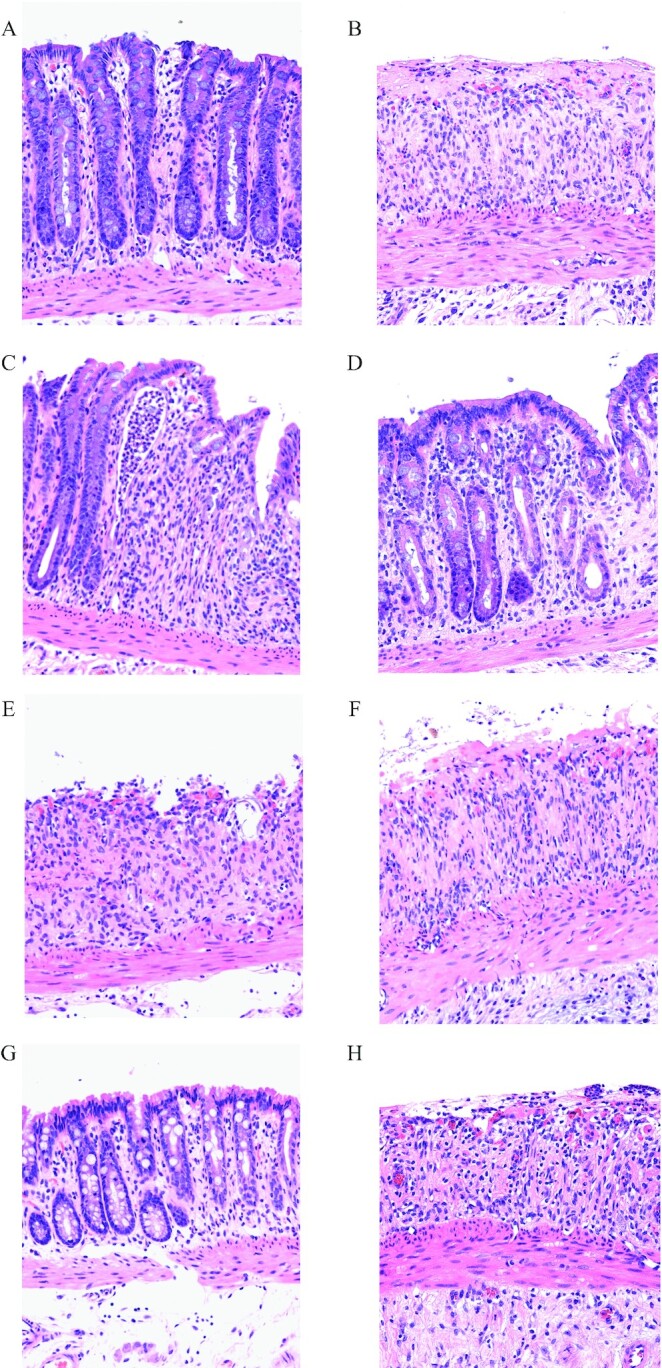
Influence of the protease inhibitors SBTI and ovomucoid on protective activity of BC + egg or EGF in DSS-induced colitis in rats. Colonic morphology of same rats as in Figure 2. (A) Normal (no DSS); (B) DSS alone; (C) DSS + BC + egg; (D) DSS + BC + egg + ovomucoid; (E) DSS + ovomucoid; (F) DSS + EGF; (G) DSS + EGF + SBTI; (H) DSS + SBTI. BC, bovine colostrum; DSS, dextran sodium sulfate; EGF, epidermal growth factor; SBTI, soybean trypsin inhibitor.

Morphology showed that, compared with normal (no DSS) controls (**Figure 3**A), administration of DSS caused almost complete loss of normal crypt structure combined with major infiltration of inflammatory cells (Figure 3B). Treatment with ovomucoid alone (Figure 3E), SBTI alone (Figure 3H), or EGF alone (Figure 3F) did not influence the damaging effect of DSS. Improvement was seen in rats that received BC + egg, with the inflammatory infiltrate being less marked and crypt structure partially maintained (Figure 3C). Rats that received the combination of BC + egg + ovomucoid (Figure 3D) or EGF + SBTI (Figure 3G) showed major improvement, with minimal inflammatory infiltrate and maintenance of crypt structures.

Formal histological scoring (Figure 2D) showed results similar to those seen on morphology; no beneficial effect was found using ovomucoid alone, SBTI alone, or EGF alone. Significant improvement was seen using BC + egg (*P* < 0.05 vs. DSS alone), with the greatest improvement seen in rats that had received either BC + egg + ovomucoid or EGF + SBTI (*P* < 0.01 vs. DSS alone).

## Discussion

We used well-validated in vitro and in vivo models to investigate the susceptibility of BC, egg, and EGF to digestion from gastric and pancreatic enzymes and examined the potential benefit of co-administering serine protease inhibitors.

The use of gastrointestinal cell lines to determine relative proliferative bioactivity of different compounds and to show changes in bioactivity following proteolytic cleavage has been used by us and others previously. For example, we showed that EGF is clipped from a 1–53 to a 1–49 form in the presence of HCl/pepsin, resulting in reduced bioactivity (10). The gastric cell line AGS was chosen as it is of human origin, expresses EGFR, correlates well with results testing BC using intestinal or colonic cell lines (8), and remains viable in the presence of pancreatic proteolytic enzymes.

Proliferative activities of BC + egg and EGF were all reduced by the sequential exposure to HCl/pepsin followed by chymotrypsin and trypsin. EGF alone showed the greatest susceptibility to digestion and is probably due to BC and egg naturally containing factors that offer partial protection against digestion. Ovomucoid was chosen as one of the test protease inhibitors as it is naturally present in egg white (11% of total protein). Ovomucoid has a molecular weight of 28 kDa and contains 3 Kazal serine trypsin inhibitor active sites within its sequence (21). BC contains several protease inhibitors, including α_2_-macroglobulin, α_2_-antiplasmin, antithrombin III, C1-inhibitor, inter-α-trypsin inhibitor, bovine plasma elastase inhibitor, and bovine plasma trypsin inhibitor, with total protease inhibitor content being much higher in BC than in the subsequent mature milk (22). Bovine plasma trypsin inhibitor contains a single Kazal inhibitor site (23). SBTI was chosen as the second test protease inhibitor, rather than bovine trypsin inhibitor, due to SBTI being readily available commercially and with a low cost to manufacture. SBTI and ovomucoid showed similar trypsin inhibitory activity, with BC and egg having 1/100th and 1/20th of the activity, respectively. SBTI and ovomucoid were both tested to show the generalizability of the findings. If progressed to clinical trials, it also allows an alternative should the patient have an allergy to one of the products.

The addition of SBTI alone to AGS cells did not stimulate proliferation, whereas a pro-proliferative effect was seen when ovomucoid was added alone, as reported by us previously (7). In most of the experimental conditions, neither ovomucoid nor SBTI inhibited the proteolytic damage of BC, egg, or EGF caused by HCl/pepsin. This was expected as pepsin has an aspartic acid active site rather than a serine active site, which is present in chymotrypsin and trypsin. There was, however, an unexpected finding of reduced proteolytic digestion by HCl/pepsin when egg + BC + ovomucoid or EGF + SBTI were combined. Several isoforms of pepsin exist that differ slightly in their preferred substrate specificity and pH profile. We previously demonstrated that most of the peptic activity is prevented if gastric juice pH is raised above 4, as occurs in patients taking proton pump inhibitors and that this rise in pH is sufficient to prevent partial cleavage of EGF within gastric juice (10). It was for this reason that all rats undergoing the DSS study received sodium bicarbonate.

UC is a chronic relapsing disease, where powerful immunosuppressive therapies are often required but have serious side effects such as immune suppression and increased risk of infections. Novel therapies are therefore required. Several different models of colitis are available; these include administering noxious compounds either orally or rectally (inducible-colitis models) or using genetically modified mice that have excess immune responses (spontaneous-colitis models). We used orally administered DSS as it is a well-established model that we and others have used to examine potential biological therapeutic agents (7, 20). An advantage of using the DSS model is that researchers can accurately decide on the temporal relation between administering test product and the induction of colitis. Innate immune mechanisms probably play an important role in mediating injury caused by DSS. Changes in both T-helper (Th) 1 and Th2 cytokine profiles occur, although the Th1 response predominates (24). To allow a reasonable number of rats and groups to be compared within a single experiment, we focused on 2 main areas:

1. The efficacy of the BC + egg combination in reducing DSS-induced injury in the presence and absence of ovomucoid. This decision was based on the facts that we have previously shown synergistic responses in reducing DSS-induced injury when BC and egg are combined (7), ovomucoid is naturally present in egg, and additional benefits were seen using BC + egg + ovomucoid in preserving against HCl/pepsin digestion.
2. The efficacy of EGF in reducing DSS-induced injury in the presence and absence of SBTI. EGF was used as an exemplar recombinant growth factor because it has been shown to be beneficial when administered systemically in DSS models (20) and in a clinical trial for UC when administered via enema (9). In addition, it is a major contributor to the pro-proliferative activity of BC, and SBTI showed additional benefits in reducing HCl/pepsin loss of bioactivity when combined with the EGF in the in vitro studies.

Results from the in vivo study showed consistent results across all the measured parameters of DSS-induced injury. Neither trypsin inhibitor improved damage when given on their own but significantly reversed weight loss and improved DAI scores and histology and MPO values when given with the BC + egg (ovomucoid) or with EGF (SBTI). Proliferation rate within the colonic crypts could not be assessed due to the almost complete loss of structure in many of the groups.

BC and eggs are important dietary source of calories, protein, fats, and minerals. In addition to their nutritional value, BC and egg both contain multiple bioactive molecules that can stimulate repair [e.g., EGF, TGF-β, insulin-like growth factor I (IGF-I) in BC, and ovomucoid and ovalbumin in egg]. They also contain factors that influence antioxidant and immune function [e.g., IL-1β, IL-6, IL-10, TNF-α in BC, lysozyme (present in both BC and egg), and ovotransferrin, phosvitin ovalbumin, and egg yolk vitellogenin in egg]. It is, therefore, likely that >1 molecule was responsible for the beneficial effects seen. For a useful review of bioactive constituents of BC, see reference (3), and for egg, see reference (11).

Although caution needs to be shown when extrapolating from animal models to humans, our studies have potential clinical relevance. Recombinant growth factors are now regularly used in clinical practice. Examples include insulin, erythropoietin, and granulocyte colony-stimulating factor. Clinical administration of growth factors for “hollow organ” gut conditions is, however, at a less well-developed stage. Glucagon-like peptide-2 (GLP-2) administered systemically increases gut growth in subjects with “short bowel syndrome” and is clinically available, although the cost is high (25). Systemic treatment with EGF has also shown promising results for treating neonatal necrotizing enterocolitis (26). There is, however, concern regarding the use of systemic growth factors for prolonged periods due to the potential risk of enhancing development of cancer in premalignant lesions (27). EGF receptors are present throughout the human gastrointestinal tract but are normally restricted to basolateral membranes (28). However, at sites of injury, luminal EGF can access these receptors, as demonstrated by the finding that EGF-containing enemas were effective in healing distal ulcerative colitis (9). Luminally administered nutraceuticals can, therefore, reach basolateral growth factor receptors in damaged areas without the risks of systemic exposure.

Topical therapy via enema administration may be useful in patients with inflammation restricted to the distal (left side) of the colon, but this method is unable to reach more proximal areas of the colon. Orally administered pro-reparative factors can reach the entire gastrointestinal tract (if prevented from digestion) and the level of purity required is less than if given intravenously, significantly reducing costs of production.

There is currently a demand from the public for more “natural” types of products, which are usually considered as “alternative therapy,” but which can possess potent biological activity. BC and egg fall into this category and have the advantage over the use of a single pharmaceutical compound (or single recombinant growth factor) of possessing multiple pro-reparative and anti-inflammatory factors, which may act synergistically (7). These products are often termed nutraceuticals (from nutrition and pharmaceuticals). Our studies suggest that SBTI and ovomucoid should also be considered within the nutraceutical category because, although they have limited activity when used in isolation, they induce synergistic responses when used with other factors. Preservation strategies to enhance efficacy of orally delivered peptides could therefore include acid suppressants to reduce HCl/pepsin digestion in the stomach (10), trypsin inhibitors such as ovomucoid or SBTI (to reduce digestion in the small intestine by pancreatic proteases), or site-specific release formulations (dependent on specific disease location).

In conclusion, our studies suggest that co-administration of trypsin inhibitors such as ovomucoid or SBTI could enhance the therapeutic efficacy of orally delivered reparative peptides when given as single factors, such as EGF, or natural combination nutraceuticals, such as BC. In addition to any intrinsic proliferative/reparative activity, as seen with ovomucoid, these trypsin inhibitors probably mediate their effects through reducing intraluminal digestion of co-administered growth factors. This approach may have value for multiple gastrointestinal conditions, including NSAID-induced small intestinal gut injury (as acid suppressants do not influence small intestinal damage), UC (colonic injury), and Crohn's disease (which predominantly affects the terminal small intestine). Clinical trials appear to be justified.

## Data Availability

Data described in the manuscript, code book, and analytic code will be made available upon request.

## Notes

Partially funded by Pantheryx Inc. The funder provided support in the form of salaries for authors (RJP, SJMtB) and provided partial funding for TM in her role as an external consultant. The specific roles of these authors are articulated in the Acknowledgments section.

Author disclosures: RJP and SJMB were paid employees of Pantheryx and TM was a paid external consultant at the time these studies were undertaken. Pantheryx Inc. is a bovine colostrum supplier to both the US and European markets. Data included in this publication form part of US Provisional Application Serial No. 63/154495 entitled Stabilized colostrum compositions of which RJP is a named inventor. This does not alter our adherence to the journals policies on sharing data and materials. Pantheryx did not have any additional role in the study design, data collection and analysis, decision to publish, or preparation of the manuscript.

Abbreviations used: AGS, human gastric adenocarcinoma cell line; BC, bovine colostrum; CT, pepsin and HCl digested followed by chymotrypsin- and trypsin-digested samples; DAI, disease activity index; DSS, dextran sodium sulfate; EGF, epidermal growth factor; EGFR, epidermal growth factor receptor; IBD, inflammatory bowel disease; MPO, myeloperoxidase; NSAID, nonsteroidal anti-inflammatory drug; P, pepsin and HCl digested samples; SBTI, soybean trypsin inhibitor; TGF, transforming growth factor; Th, T-helper; TNBS, trinitrobenzenesulfonic acid; U, undigested samples; UC, ulcerative colitis.

## References

[1] Godat S, Fournier N, Safroneeva E, Juillerat P, Nydegger A, Straumann A, Vavricka S, Biedermann L, Greuter T, Fraga M et al.; Swiss IBD Cohort Study Group. Frequency and type of drug-related side effects necessitating treatment discontinuation in the Swiss Inflammatory Bowel Disease Cohort. Eur J Gastroenterol Hepatol. 2018;30:612–20. 29384798 10.1097/MEG.0000000000001078

[2] Forbes A, Escher J, Hébuterne X, Kłęk S, Krznaric Z, Schneider S, Shamir R, Stardelova K, Wierdsma N, Wiskin AE et al. ESPEN guideline: clinical nutrition in inflammatory bowel disease. Clin Nutr. 2017;36:321–47. 28131521 10.1016/j.clnu.2016.12.027

[3] Playford RJ, Weiser MJ. Bovine colostrum: its constituents and uses. Nutrients. 2021;13:265. 33477653 10.3390/nu13010265 PMC7831509

[4] Playford RJ, Macdonald CE, Calnan DP, Floyd DN, Podas T, Johnson W, Wicks AC, Bashir O, Marchbank T. Co-administration of the health food supplement, bovine colostrum, reduces the acute non-steroidal anti-inflammatory drug-induced increase in intestinal permeability. Clin Sci. 2001;100:627–33. 11352778

[5] Marchbank T, Davison G, Oakes JR, Ghatei MA, Patterson M, Moyer MP, Playford RJ. The nutriceutical bovine colostrum truncates the increase in gut permeability caused by heavy exercise in athletes. Am J Physiol Gastrointest Liver Physiol. 2011;300: G477–84. 21148400 10.1152/ajpgi.00281.2010

[6] Filipescu IE, Leonardi L, Menchetti L, Guelfi G, Traina G, Casagrande-Proietti P, Piro F, Quattrone A, Barbato O, Brecchia G. Preventive effects of bovine colostrum supplementation in TNBS-induced colitis in mice. PLoS One. 2018;13:e202929. 10.1371/journal.pone.0202929 PMC6107273 30138385

[7] Playford RJ, Garbowsky M, Marchbank T. Pasteurized chicken egg powder stimulates proliferation and migration of AGS, RIE1, and Caco-2 cells and reduces NSAID-induced injury in mice and colitis in rats. J Nutr. 2020;150:1434–42. 32286629 10.1093/jn/nxaa083

[8] Playford RJ, Cattell M, Marchbank T. Marked variability in bioactivity between commercially available bovine colostrum for human use; implications for clinical trials. PLoS One. 2020;15:0234719. 10.1371/journal.pone.0234719 PMC7299325 32555629

[9] Khan Z, Macdonald C, Wicks AC, Holt MP, Floyd D, Ghosh S, Wright NA, Playford RJ. Use of the “nutriceutical”, bovine colostrum, for the treatment of distal colitis: results from an initial study. Aliment Pharmacol Ther. 2002;16:1917–22. 12390100 10.1046/j.1365-2036.2002.01354.x

[10] Playford RJ, Marchbank T, Calnan DP, Calam J, Royston P, Batten JJ, Hansen HF. Epidermal growth factor is digested to smaller, less active forms in acidic gastric juice, Gastroenterology. 1995;108:92–101. 7806067 10.1016/0016-5085(95)90012-8

[11] Réhault-Godbert S, Guyot N, Nys Y. The golden egg: nutritional value, bioactivities, and emerging benefits for human health. Nutrients. 2019;11:684. 10.3390/nu11030684 PMC6470839 30909449

[12] Savage GP, Morrison SC. Trypsin inhibitors. In: Caballero B, editor. Encyclopedia of food sciences and nutrition. 2nd ed. Cambridge, Massachusetts, USA: Academic Press; 2003. p. 5878–84.

[13] Erlander BF, Kokowsky N, Cohen. The preparation and properties of two new chromogenic substrates of trypsin, Arch Biochem Biophys. 1961;95:271–8. 13890599 10.1016/0003-9861(61)90145-x

[14] Barranco SC, Townsend CM Jr, Casartelli C. Establishment and characterization of an in vitro model system for human adenocarcinoma of the stomach. Cancer Res. 1983;43:1703–9. 6831414

[15] Marchbank T, Weaver G, Nilsen-Hamilton M, Playford RJ. Pancreatic secretory trypsin inhibitor is a major motogenic and protective factor in human breast milk. Am J Physiol Gastrointest Liver Physiol. 2009;296:G697–703. 19147803 10.1152/ajpgi.90565.2008

[16] Ha X, Peng J, Zha H, Deng Z, Dong J, Fan H, Zhao Y, Li B, Feng Q, Yang Z. Enhancement of gastric ulcer healing and angiogenesis by hepatocyte growth factor gene mediated by attenuated salmonella in rats. J Korean Med Sci. 2017;32:186–94. 28049228 10.3346/jkms.2017.32.2.186 PMC5219983

[17] Hung CR, Cheng JT. Betel quid chewing damaged gastric mucosa: protective effects of cimetidine and sodium bicarbonate. Chin J Physiol. 1994;37:213–8. 7796637

[18] Cooper HS, Murthy SN, Shah RS, Sedergran DJ. Clinicopathologic study of dextran sulfate sodium experimental murine colitis. Lab Invest. 1993;69;238–49. 8350599

[19] Williams KL, Fuller CR, Dieleman LA, DaCosta CM, Haldeman KM, Sarto RB, Lund PK. Enhanced survival and mucosal repair after dextran sodium sulfate-induced colitis in transgenic mice that overexpress growth hormone. Gastroenterology. 2001;120:925–37. 11231946 10.1053/gast.2001.22470

[20] FitzGerald AJ, Pu M, Marchbank T, May FE, Boyle J, Yadollahi-Farsani M, Ghosh S, Playford RJ. Synergistic effects of systemic trefoil factor family 1 (TFF1) peptide and epidermal growth factor in a rat model of colitis. Peptides. 2004;25:793–801. 15177874 10.1016/j.peptides.2003.12.022

[21] Saxena I, Tayyab S. Protein proteinase inhibitors from avian egg whites. Cell Mol Life Sci. 1997;53:13–23. 9117993 10.1007/PL00000575 PMC11147361

[22] Godden SM, Lombard JE, Woolums AR. Colostrum management for dairy calves. Vet Clin North Am Food Anim Pract. 2019;35:535–56. 31590901 10.1016/j.cvfa.2019.07.005 PMC7125574

[23] Zhou JM, Liu C, Tsou CL. Kinetics of trypsin inhibition by its specific inhibitors. Biochemistry. 1989;28:1070–6. 2713358 10.1021/bi00429a022

[24] Egger B, Bajaj-Elliott M, MacDonald TT, Inglin R, Eysselein VE, Büchler MW. Characterisation of acute murine dextran sodium sulphate colitis: cytokine profile and dose dependency. Digestion. 2000;62:240–8. 11070407 10.1159/000007822

[25] Jeppesen PB. Clinical significance of GLP-2 in short-bowel syndrome. J Nutr. 2003;133:3721–4. 14608103 10.1093/jn/133.11.3721

[26] Sullivan PB, Brueton MJ, Tabara Z, Goodlad RA, Lee CY, Wright NA. Epidermal growth factor in necrotizing enterocolitis. Lancet. 1991;338:53–4. 10.1016/0140-6736(91)90042-n 1676104

[27] Bashir O, Fitzgerald AJ, Berlanga-Acosta J, Playford RJ, Goodlad RA. Effect of epidermal growth factor administration on intestinal cell proliferation, crypt fission and polyp formation in multiple intestinal neoplasia (Min) mice. Clin Sci (Lond). 2003;105: 323–30. 12749762 10.1042/CS20030023

[28] Playford RJ, Hanby AM, Gschmeissner S, Peiffer LP, Wright NA, McGarrity T. The epidermal growth factor receptor (EGF-R) is present on the basolateral, but not the apical, surface of enterocytes in the human gastrointestinal tract. Gut. 1996;39:262–6. 8977341 10.1136/gut.39.2.262 PMC1383309

